# Reduced magnetic resonance angiography signal intensity in the middle cerebral artery ipsilateral to severe carotid stenosis may be a practical index of high oxygen extraction fraction

**DOI:** 10.1007/s00330-021-08272-3

**Published:** 2021-10-12

**Authors:** Takahisa Mori, Kazuhiro Yoshioka, Yuhei Tanno, Shigen Kasakura, Yuichi Miyazaki

**Affiliations:** 1 Department of Stroke Treatment, Shonan Kamakura General Hospital, 1370-1, Kamakura City, Kanagawa, Okamoto 247-8533 Japan; 2 Department of Neurology, Nakatsugawa Municipal General Hospital, Gifu, Japan; 3 Department of Neuro-Endovascular Therapy, Saitama Medical University International Medical Center, Saitama, Japan

**Keywords:** Carotid stenosis, Magnetic resonance angiography, Middle cerebral artery, Oxygen

## Abstract

**Objectives:**

Angiographic “slow flow” in the middle cerebral artery (MCA), caused by carotid stenosis, may be associated with high oxygen extraction fraction (OEF). If the MCA slow flow is associated with a reduced relative signal intensity (rSI) of the MCA on MR angiography, the reduced rSI may be associated with a high OEF. We investigated whether the MCA slow flow ipsilateral to carotid stenosis was associated with a high OEF and aimed to create a practical index to estimate the high OEF.

**Methods:**

We included patients who underwent digital subtraction angiography (DSA) and MRA between 2015 and 2019 to evaluate carotid stenosis. MCA slow flow by image count using DSA, MCA rSI, minimal luminal diameter (MLD) of the carotid artery, carotid artery stenosis rate (CASr), and whole-brain OEF (wb-OEF) was evaluated. When MCA slow flow was associated with a high wb-OEF, the determinants of MCA slow flow were identified, and their association with high wb-OEF was evaluated.

**Results:**

One hundred and twenty-seven patients met our inclusion criteria. Angiographic MCA slow flow was associated with high wb-OEF. We identified MCA rSI and MLD as determinants of angiographic MCA slow flow. The upper limits of MCA rSI and MLD for angiographic MCA slow flow were 0.89 and 1.06 mm, respectively. The wb-OEF was higher in patients with an MCA rSI ≤ 0.89 and ipsilateral MLD ≤ 1.06 mm than patients without this combination.

**Conclusions:**

The combination of reduced MCA rSI and ipsilateral narrow MLD is a straightforward index of high wb-OEF.

**Key Points:**

*• The whole-brain OEF in patients with angiographic slow flow in the MCA ipsilateral to high-grade carotid stenosis was higher than in patients without it.*

*• Independent determinants of MCA slow flow were MCA relative signal intensity (rSI) on MRA or minimal luminal diameter (MLD) of the carotid stenosis.*

*• The wb-OEF was higher in patients with an MCA rSI ≤ 0.89 and ipsilateral MLD ≤ 1.06 mm than patients without this combination.*

**Supplementary Information:**

The online version contains supplementary material available at 10.1007/s00330-021-08272-3.

## Introduction

An increased oxygen extraction fraction (OEF) due to decreased cerebral blood flow (CBF) on PET is an independent predictor of subsequent stroke in patients with carotid artery stenosis or occlusion [1–3]. Furthermore, intracranial hemorrhage due to hyperperfusion rarely occurs after carotid revascularization in patients with cerebral hemodynamic insufficiency due to extremely high-grade carotid stenosis (hg-CS) [4]. Pre-carotid artery stenting (CAS) OEF in patients with post-CAS hyperperfusion syndrome was higher than in patients without post-CAS hyperperfusion syndrome [5]. PET scanning is impractical for routine evaluation in most facilities. SPECT imaging may be used to detect decreased CBF [6]. However, SPECT is an expensive examination involving radioisotopes, and it cannot measure the OEF. SPECT is not routinely conducted in most facilities.

Noninvasive OEF measurement using MRI at 3 T or 7 T can be substituted for PET in patients with carotid artery stenosis or occlusion [7–9], and cerebrovascular reactivity with blood oxygen level–dependent MRI at 3 T corresponds to CBF perfusion reserve measurements obtained with PET, especially for detecting hemodynamic failure [10]. However, this technology is not routinely available in most facilities. The OEF can be calculated by measuring the blood oxygen content with invasive procedures [5, 11]; however, it is not the standard in most facilities. Thus, a more feasible and practical index to identify patients with a high OEF is needed in routine practice.

The carotid artery stenosis rate (CASr), based on the North American Symptomatic Carotid Endarterectomy Trial criteria [12], is an unreliable cerebral hemodynamic status indicator. However, a significant relationship exists between the PET hemodynamic parameters and arteriographic circulation pattern [13]. Angiographic “slow flow” in the middle cerebral artery (MCA) ipsilateral to hg-CS may be associated with a high OEF [14, 15]. Post-CAS OEF decreased compared to pre-CAS OEF in patients with angiographic slow flow in the MCA distal to high-grade carotid stenosis [14]. If MCA slow flow ipsilateral to severe carotid stenosis is associated with a reduced MCA relative signal intensity (rSI) on MR angiography (MRA), the reduced MCA rSI may be associated with a high OEF, and then may be a practical index of high OEF, which would be beneficial compared to the unusual and invasive catheter-based OEF measurement. Therefore, we attempted to create a practical and feasible index to estimate a high OEF, and we investigated the relationship between MCA slow flow, OEF, MCA rSI, and carotid artery stenosis.

## Methods

For this retrospective cross-sectional study, we searched patients in the Institutional Stroke Database and included patients who (1) were admitted between January 2015 and March 2019 for elective carotid artery stenting (CAS); (2) underwent digital subtraction angiography (DSA), MRA, and SPECT before CAS; and (3) underwent blood sampling whole-brain OEF (wb-OEF) immediately before CAS [5, 9]. We excluded patients who (1) underwent CAS within 29 days of their last ischemic attack; (2) had an ipsilateral CAS history; (3) had a contraindication to MRI; (4) had no blood sampling wb-OEF examination; or (5) underwent CAS of high-grade stenosis of the common carotid artery. The data that support the findings of this study are available from the corresponding author on reasonable request.

### Angiographic slow flow in the MCA

**Fig. 1:**
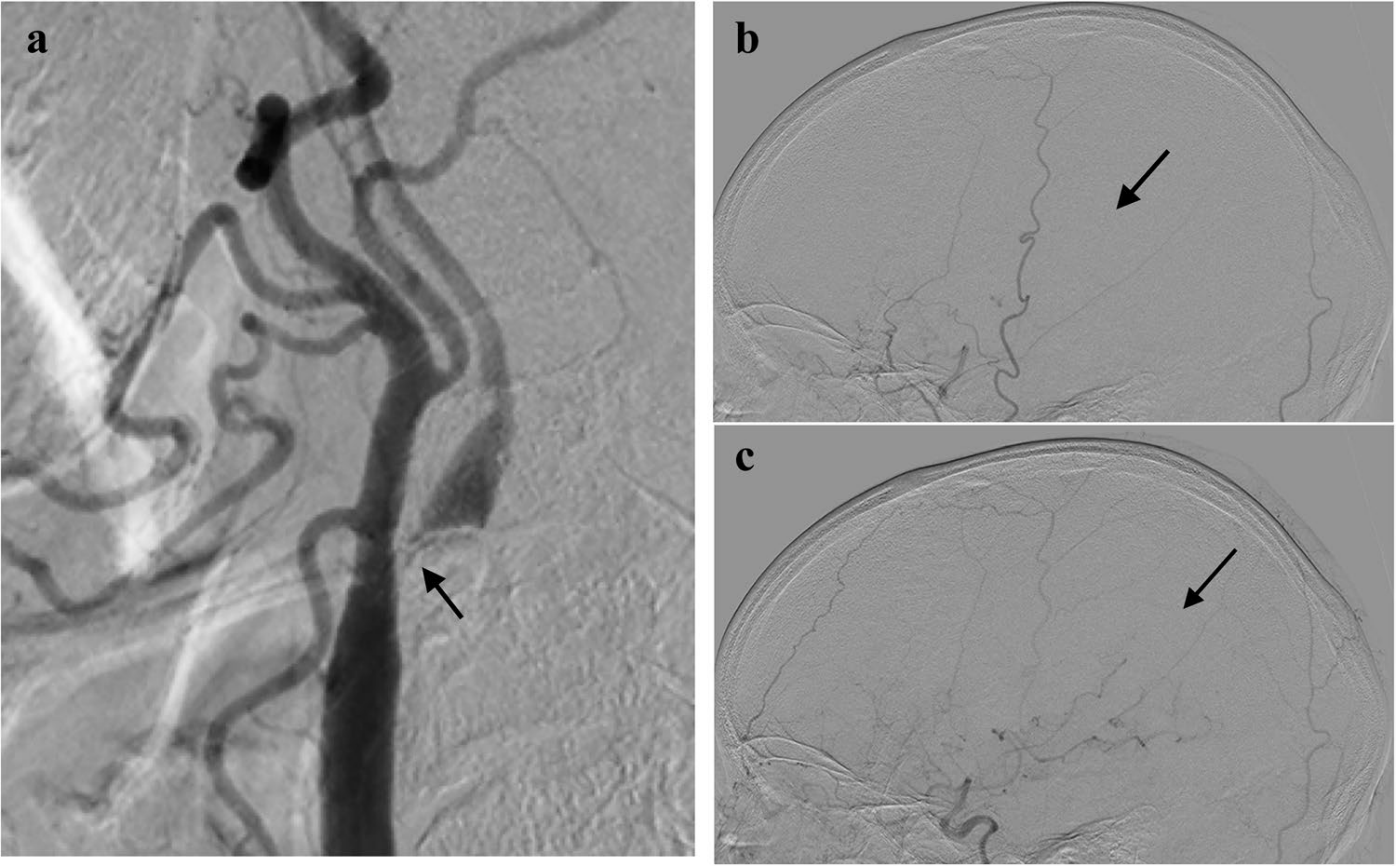
Digital subtraction angiography. **a** The carotid angiogram of the right carotid artery (lateral projection) reveals an extremely high-grade stenosis (arrow): the MLD is 0.31 mm. **b**, **c** The cerebral angiograms (lateral projection) reveal “slow flow” in the right middle cerebral artery (arrow). Image **c** is count 3, after image **b**. MLD, minimal luminal diameter

We performed DSA (INFX-8000 V with PureBrain, Canon Medical Systems) by injecting 6 mL of a nonionic contrast medium (iopamidol; 300 mg/mL) (Bayer Yakuhin, Ltd.) at a rate of 4 mL/s through a 3-French (Fr) cerebral diagnostic catheter. Image acquisition was conducted at a rate of 3 images/s. We defined angiographic “slow flow” in the MCA due to a hg-CS on DSA as delayed maximal filling of the contrast medium in the MCA peripheral branches, compared to that of the ipsilateral external carotid circulation (Fig. 1) [15]. When compared to the maximal filling in the ipsilateral external carotid circulation, we defined a delay of zero image until the maximal filling in the MCA branches as count 0 (i.e., no delay), a delay of one image as count 1, a delay of two images as count 2, and so on (Fig. 1c).

### MCA rSI

**Fig. 2:**
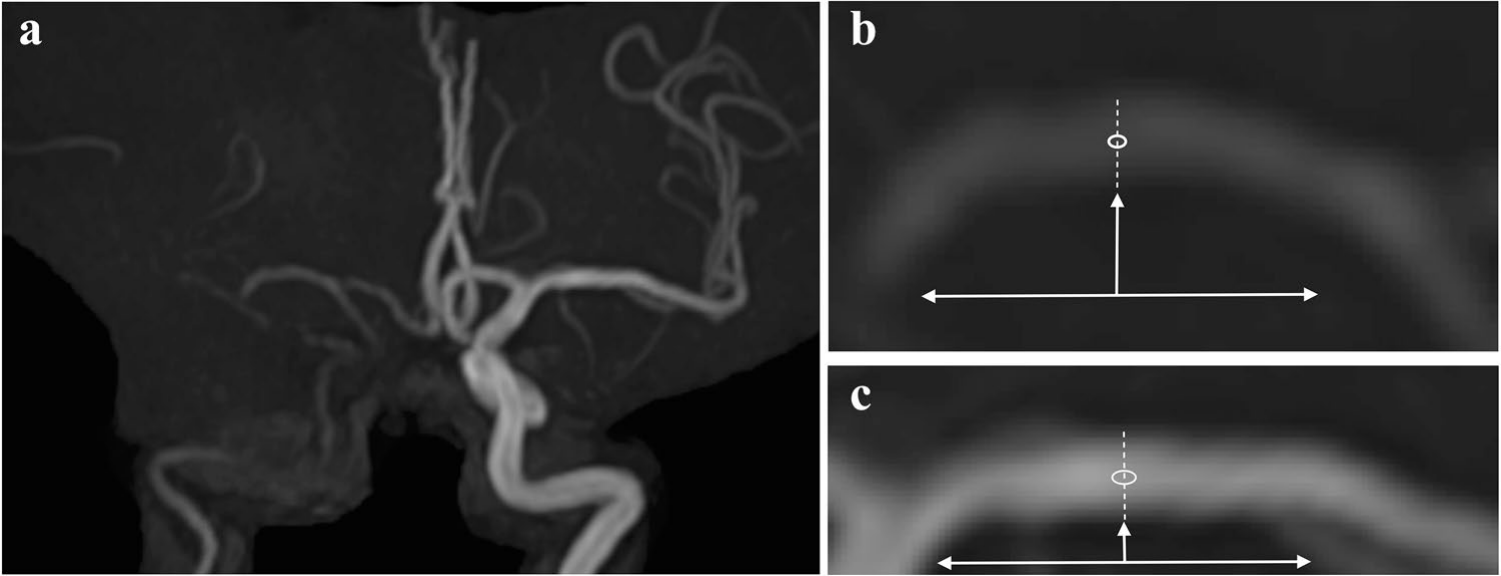
Magnetic resonance angiography of the same patient in Fig. 1. **a** MRA findings (anterior–posterior view). **b** A zoomed-in image of the right MCA M1 segment. The horizontal two-headed arrow shows the M1 segment. The vertical arrow indicates the midportion of the M1 segment. The dotted line shows the diameter of the midportion. A small circle is set in the center of the dotted line. **c** A zoomed-in image of the left MCA M1 segment. The horizontal two-headed arrow shows the M1 segment. The vertical arrow indicates the midportion of the M1 segment. The dotted line indicates the diameter of the midportion. A small circle is set in the center of the dotted line. The MCA rSI is 0.42 (i.e., 962 [circle in **b**] divided by 2287 [circle in **c**]). MCA, middle cerebral artery; MRA, magnetic resonance angiography; rSI, relative signal intensity

We conducted MRA using a 3-T MRI machine (Achieva 3.0 T X Quasar; Philips Japan, Ltd.), which was equipped with an eight-channel head coil. Three-dimensional time-of-flight MRA was conducted using the three-dimensional fast field-echo sequence. The parameters were as follows: repetition time, 24 ms; echo time, 3.45 ms; field of view, 220 × 198 × 169.2 mm^3^; flip angle, 17°; matrix size, 480 × 704; slice thickness, 1.2 mm; and scan time, 3.20 min. The MRA image (anterior–posterior view) was zoomed in, and small circular regions of interest were set on the center portions at the midportions in the bilateral MCA M1 segments. The signal intensity of the regions of interest was measured, and the MCA rSI was defined as follows (Fig. 2): (signal intensity on the affected side M1)/(signal intensity on the contralateral M1).

#### SPECT

**Fig. 3:**
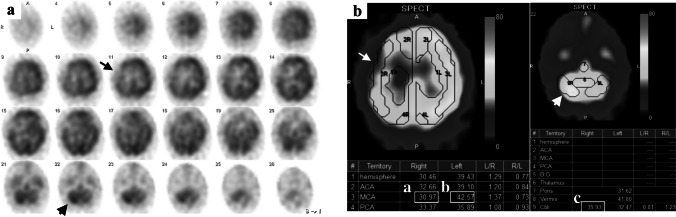
Single-photon emission computerized tomography of the same patient in Fig. 1. **a**, **b** SPECT reveals decreased rCBF in the right MCA territory (arrow) and the rCBF in the right cerebellum (arrowhead). **b** The CBF analysis software reveals an AI% of 73% (i.e., 30.97 (a) divided by 42.57 (b)) and an rCBF% of 78.7% (i.e., 30.97 (a) divided by 39.93 (c)). AI%, asymmetric index; CBF, cerebral blood flow; MCA, middle cerebral artery; rCBF, regional cerebral blood flow; SPECT, single-photon emission computerized tomography

We conducted SPECT (E-CAM, Siemens Healthineers) and measured CBF 1 week before CAS using a rotating dual-head gamma camera with a low-energy high-resolution collimator. We measured CBF with ^123^I-labeled N–isopropyl-p-iodoamphetamine (^123^I-IMP) (Nihon Medi-Physics Co., Ltd.) in combination with the graph plot method [16]. A dose of 185 MBq ^123^I-IMP was infused rapidly into the right cubital vein. Chest and head dynamic planar images were acquired with a 128 × 128 matrix at 2 s per frame for 60 frames, beginning a few seconds before the transvenous infusion of ^123^I-IMP. We subsequently obtained head SPECT images on a 64 × 64 matrix beginning 15 min after the ^123^I-IMP injection and continued for 22 min. Regions of interest were automatically set to measure the regional CBF (rCBF) in the SPECT images using a CBF-analyzing software (Neuro Flexor, Canon Medical Systems) implanting the protocol of a previous report (Fig. 3a) [17].

We used two common indices for qualitative evaluation of relative CBF: (1) the asymmetric index (AI%) [18] and (2) the percentage of rCBF (rCBF%) [19].The AI% was defined as the rCBF ratio of the affected MCA territory versus the contralateral hemisphere. It was calculated as follows (Fig. 3b): (rCBF in the affected MCA territory/rCBF in the contralateral MCA territory) × 100 (%).

The rCBF% was defined as the rCBF ratio of the affected MCA territory versus the ipsilateral cerebellum. It was calculated as follows (Fig. 3b): (rCBF in the affected MCA territory/rCBF in the ipsilateral cerebellum) × 100 (%).

### Whole-brain OEF

We positioned a 5-Fr temporary bipolar pacing catheter in the right ventricle through the femoral vein to prevent bradycardia or cardiac arrest during CAS. Before positioning the temporary pacing catheter, we introduced a 4-Fr catheter (Cerulean, Medikit Co., Ltd.) into the dominant-side jugular bulb via transfemoral venous access and sampled blood with heparinized syringes (2.5 mL). We sampled blood in the carotid artery through the arterial guide catheter (Online Figure I) [14, 19]. We measured the arterial oxygen content and venous oxygen content with a blood gas analyzer and calculated the wb-OEF to estimate the risk of hyperperfusion syndrome after CAS [5]. The calculation of the wb-OEF was as follows: wb-OEF = (arterial oxygen content − venous oxygen content)/arterial oxygen content.

### Evaluation

Before CAS, we evaluated the patients’ characteristics, delay of the MCA filling ipsilateral to hg-CS, CASr, and minimal luminal diameter (MLD) on angiographic images, MCA rSI on MRA, ultrasonographic peak systolic velocity (PSV) of carotid stenosis, rCBF% and AI%, and wb-OEF. We compared the wb-OEF in patients with count ≥ 1 with that of patients with count 0 (i.e., no delay), and identified the count that resulted in a high wb-OEF. We selected the MCA rSI, CASr, MLD, and PSV as probable predictors of slow flow in the MCA. We compared the differences in MCA rSI, CASr, MLD, and PSV between patients with values less than the count of high wb-OEF (i.e., Group 0) and patients with values greater than or equal to the count (i.e., Group I). We evaluated the correlations between the MCA rSI, CASr, MLD, and PSV. We identified the independent predictors among probable factors of slow flow to distinguish patients in Group I from patients in Group 0 and estimated the predictors’ upper limit values needed to distinguish patients in Group I from patients in Group 0. We evaluated the differences in the wb-OEF between patients with the predictive values needed for Group I and patients without them.

In cases of OEF increased due to occlusive cerebrovascular disease, the CBF decreased [20]. In cases of severe carotid stenosis causing post-carotid endarterectomy hyperperfusion, CBF ipsilateral to severe carotid stenosis decreased, and the MCA signal intensity ipsilateral to severe carotid stenosis had a correlation with ipsilateral CBF on the SPECT scans [21]. When the MCA rSI was an independent predictor for Group I, we conducted secondary analysis and calculated the correlation coefficient of the MCA rSI with the rCBF% or the AI% in patients with the predictive values needed for Group I. When the MCA rSI correlated with rCBF% or with AI%, we estimated the upper limit values of the MCA rSI for rCBF% < 90% or AI < 90% as CBF-decrease markers.

### Interrater reliability

Three raters (T.M., K.Y., Y.M.) independently measured bilateral M1 signal intensity on MRA images in randomly chosen patients and calculated MCA rSI.

### Statistical analysis

The chi-square test was used to compare the categorical variables. Non-normally distributed continuous variables are expressed as the median and the interquartile range. The Wilcoxon rank-sum test was used to compare unpaired groups. We used the multiple comparison test to compare all possible pairs between variables predicting slow flow in the MCA or between the factors of slow flow in the MCA. Spearman’s rank correlation coefficient (*r*_s_) was used to measure the relationship strength between non-normally distributed variables. We defined 0 ≤ |*r*_s_| < 0.1 as “no correlation,” 0.1 ≤ |*r*_s_| < 0.4 as “weak correlation,” 0.4 ≤ |*r*_s_| < 0.6 as “moderate correlation,” and 0.6 ≤ |*r*_s_| as “strong correlation.” Multicollinearity was defined as a strong correlation between the variables. We compared variables with a significant difference between patients with and without slow flow in the MCA. When variables were strongly correlated with one another, we adopted the variable with a larger *z* value. On excluding the variables with multicollinearity, we conducted multiple logistic regression analyses to identify independent predictors of dichotomous variables and estimated the upper limit values of the predictors by using the AUC derived from the ROC curves of the logistic regression model. Interrater reliability was measured using intraclass correlation coefficient (ICC (2,1)). A value of *p *< 0.05 was statistically significant. We used JMP software (version 15.2; SAS Institute) for statistical analyses.

## Results

One hundred and twenty-seven patients (66.5%) of 191 patients scheduled to undergo elective CAS met our inclusion criteria (Online Figure II). The patients’ characteristics are summarized in Online Tables I and II. This study included 86, 19, 9, 6, 5, and 2 patients in counts 0, 1, 2, 3, 4, and 5, respectively, in images delay until the maximal filling in the MCA. Significant differences existed in the wb-OEF between patients in count 0 and patients in count 1 or count 4 (Online Tables III and IV). Patients were divided into two groups—patients in count 0 (Group 0) and patients in counts 1–5 (Group I)—because the patients in count 1 had a greater wb-OEF than did patients in count 0 and because the number of patients in counts 2–5 was small. A significant difference in the wb-OEF existed between the two groups (*p *< 0.0001) (Table 1).

**Table 1:** The relationship between the whole-brain oxygen ejection fraction and the groups, based on the delay of the maximal filling of the contrast medium in the middle cerebral artery distal to the carotid stenosis

Group	wb-OEF, median (IQR)	*p* value
0 (*n *= 86)	0.36 (0.31–0.40)	< 0.0001
I (*n *= 41)	0.42 (0.38–0.47)	

Group 0 consists of patients with count 0; Group I consists of patients with count 1, 2, 3, 4, or 5. *IQR*, interquartile range; *wb-OEF*, whole-brain oxygen extraction fraction; *p*, probability

The MCA rSI, MLD, CASr, and PSV were significantly different between Group 0 and Group I; however, no differences in rCBF% or AI% existed between Group 0 and Group I (Online Table V). The MLD and CASr were strongly correlated (Online Table VI). After excluding CASr with multicollinearity, we conducted logistic regression analysis for Group I by using the variables MCA rSI, MLD, and PSV, and identified MCA rSI and MLD as the independent predictors in Group I (Table 2). The ROC curves estimated the upper limit values of MCA rSI and MLD for Group I as 0.89 (AUC, 0.823) and 1.06 mm (AUC, 0.782), respectively (Online Table VII). Among the 60 patients with an MCA rSI > 0.89 and MLD > 1.06 mm, six patients belonged to Group I. Among the 30 patients with MCA rSI ≤ 0.89 and MLD > 1.06 mm, or with MCA rSI > 0.89 and MLD ≤ 1.06 mm, eight patients belonged to Group I (odds ratio: 3.3). Among 37 patients with an MCA rSI ≤ 0.89 and MLD ≤ 1.06 mm, 27 patients belonged to Group I (odds ratio, 24.3). The combination of MCA rSI ≤ 0.89 and MLD ≤ 1.06 mm was a predictor of being in Group I (Online Table VIII). The wb-OEF was significantly different, based on whether the combination of MCA rSI and MLD was or were not at the upper limits of 0.89 and 1.06 mm, respectively (Online Table IX). The wb-OEF was higher in patients (*n *= 37) with the combination of MCA rSI ≤ 0.89 and MLD ≤ 1.06 mm than in patients (*n *= 60) with the combination of MCA rSI > 0.89 and MLD > 1.06 (Online Table X).

**Table 2:** Results of multiple logistic regression analysis to identify independent predictors of Group I

	*N*	OR	*p* value	AUC	BIC
	127		< 0.0001	0.845	131
MCA rSI		7.38E − 4 (1.04E − 5 to 2.81E − 2)	0.0003		
MLD		0.310 (0.108–0.761)	0.0172		
PSV		0.999 (0.995–1.00)	0.7806		

Group I consists of patients with count 1, 2, 3, 4, or 5. *AUC*, area under the curve; *BIC*, Bayesian information criterion; *MCA rSI*, middle cerebral artery relative signal intensity; *MLD*, minimum luminal diameter; *N*, number; *OR*, odds ratio; *p*, probability; *PSV*, peak systolic velocity

In 37 patients with MCA rSI ≤ 0.89 and MLD ≤ 1.06 mm, the MCA rSI was moderately correlated with the rCBF% and the AI%. In addition, the correlation coefficient was larger between the MCA rSI and AI% than between the MCA rSI and rCBF% (Online Table XI). The ROC curves indicated that the estimated upper limits of MCA rSI were 0.69 for rCBF% < 90% (AUC, 0.671) and 0.71 for AI% < 90% (AUC, 0.922) (Online Tables XII and XIII).

### Interrater reliability

We found that the interrater reliability was substantial for MCA rSI after measuring bilateral MCA signal intensity on MRA images (ICC (2,1) = 0.728, *n *= 24, three raters).

## Discussion

This study aimed to create a more straightforward index to estimate a high OEF and found that the combination of MCA rSI ≤ 0.89 and ipsilateral hg-CS with an MLD ≤ 1.06 mm was the predictor of high wb-OEF. In patients with this combination, the upper limit value of the MCA rSI was 0.71 for AI% < 90% on SPECT scans. Decreased CBF and high wb-OEF could be estimated by combining the MCA rSI on MRA and MLD on DSA without SPECT.

Hyperperfusion syndrome occurs after the revascularization of a hg-CS that has caused hemodynamic insufficiency [15]. However, in a carotid endarterectomy survey, the risk of hyperperfusion syndrome was routinely evaluated without SPECT by only 102 (15.5%) of 664 anesthesiologists [22]. MCA rSI and MLD are readily available, and the index can be practically and feasibly applied in routine practice. In 1948, Kety and Schmidt measured the arterial oxygen content and venous oxygen content in the internal jugular vein [9]. They reported an arterial oxygen content of 16.8 vol.% and jugular vein oxygen content of 10.7 vol.% under 21% oxygen inhalation, indicating a wb-OEF of 0.36. In addition, a previous study reported a wb-OEF of 0.37 ± 0.4 [23]. The wb-OEF of 0.38 (0.33–0.42) in the present study was nearly the same as that reported in previous studies [9, 23]. DSA, jugular vein blood sampling, and SPECT with radioisotopes used in the present study are invasive examinations. However, the use of blood sampling in the jugular vein or SPECT would no longer be needed because the combination of MCA rSI on MRA and the MLD on DSA could be used to identify patients with high wb-OEF. Carotid computed tomography angiography can be used to measure the MLD in carotid artery stenosis [24], and the MLD may be measured without DSA. Cerebral hemodynamic insufficiency can be more easily estimated using noninvasive examinations.

Our study had several limitations, which include the small sample size of patients. It was a retrospective, cross-sectional observational study conducted in a single institution. The sequence and parameters of three-dimensional time-of-flight MRA must be standardized. Standardization is required to set the regions of interest on MRA. The utility of reduced MCA rSI in patients with severe MCA stenosis was not investigated. It was not investigated whether the MCA rSI was reduced and the wb-OEF was high in patients with sufficient collateral flow to the MCA ipsilateral to severe carotid stenosis causing angiographic MCA slow flow. The critical value of reduced MCA rSI ipsilateral to severe carotid stenosis to predict a subsequent stroke has not been investigated. A prospective study in multiple facilities is warranted to confirm our results.

In conclusion, the combination of MCA rSI ≤ 0.89 on MRA and ipsilateral hg-CS with an MLD ≤ 1.06 mm is a practical and straightforward index of wb-OEF increase. Furthermore, when the MCA rSI is ≤ 0.71, the AI% on SPECT may be < 90%. This index may be useful to identify patients with hemodynamic insufficiency in routine practice.

## Supplementary Information

Below is the link to the electronic supplementary material.

Supplementary file1 (PDF 330 KB)

## Abbreviations

^123^I-IMP: ^123^I-labeled N-isopropyl-p-iodoamphetamine
AI: Asymmetric index
CAS: Carotid artery stenting
CASr: Carotid artery stenosis rate
CBF: Cerebral blood flow
DSA: Digital subtraction angiography
hg-CS: High-grade carotid stenosis
MCA: Middle cerebral artery
MLD: Minimal luminal diameter
MRA: Magnetic resonance angiography
OEF: Oxygen extraction fraction
PSV: Peak systolic velocity
rCBF: Regional cerebral blood flow
rSI: Relative signal intensity
wb-OEF: Whole-brain oxygen extraction fraction

## Declarations

All procedures described in the study were performed in accordance with the ethical standards of the institution (Shonan Kamakura General Hospital) and with the 1964 Helsinki Declaration.

### Guarantor

The scientific guarantor of this publication is Takahisa Mori, MD. PhD.

### Statistics and biometry

No complex statistical methods were necessary for this paper.

### Methodology

• retrospective

• cross-sectional study

• performed at one institution
