# Supplementary material for: Reduced magnetic resonance angiography signal intensity in the middle cerebral artery ipsilateral to severe carotid stenosis may be a practical index of high oxygen extraction fraction
Source: Eur Radiol. 2021 Oct 12;32(3):2023–9. doi: 10.1007/s00330-021-08272-3 (PMC8831255; doi:10.1007/s00330-021-08272-3)
Supplement: Supplementary file 1 — Supplementary file1 (PDF 330 KB) [file 330_2021_8272_MOESM1_ESM.pdf]

SUPPLEMENTAL MATERIAL

Reduced Magnetic Resonance Angiography Signal Intensity in the Middle Cerebral Artery Ipsilateral to Severe Carotid Stenosis may be a Practical Index of High Oxygen Extraction Fraction

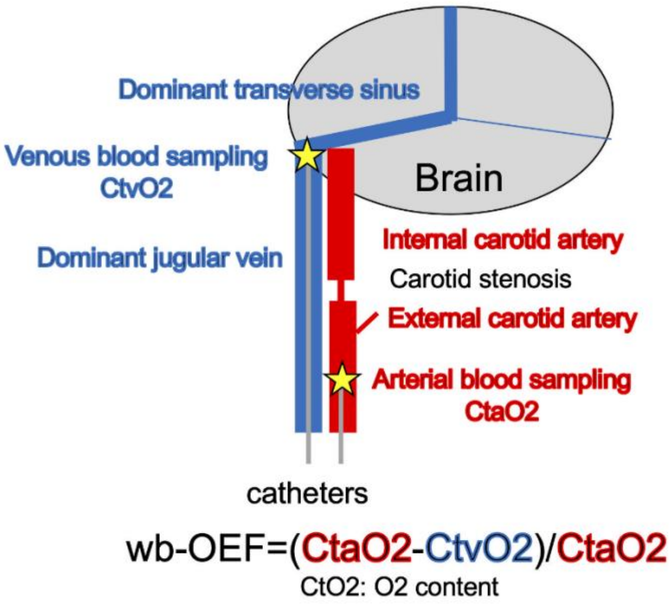

Figure I. Schematic of the arterial and venous blood sampling method.

The yellow star indicates the blood sampling point. Red represents the arterial blood flow and blue represents the venous blood flow.

CtO2, oxygen content; CtaO2, arterial oxygen content; CtvO2, venous oxygen content; O2, oxygen; wb-OEF, whole-brain oxygen extraction fraction

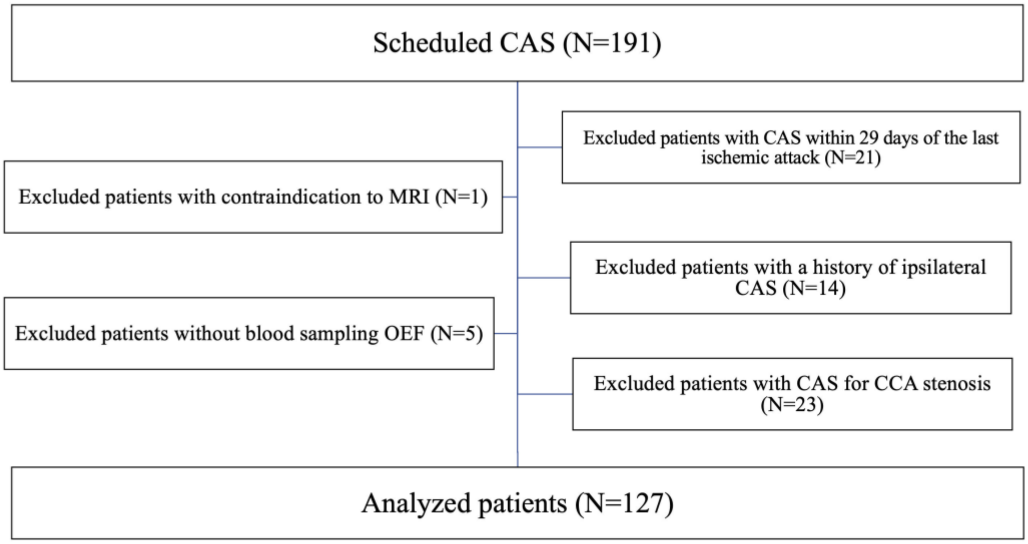

Figure II. Flow chart of the patient selection method for the analysis.
CAS, carotid artery stenting, CCA, common carotid artery, MRI, magnetic resonance imaging; OEF, oxygen extraction fraction

Table I. Characteristics of the overall patients (n=127)

Characteristic	Finding
Age (y)	77 (73-81)
Male sex, n (%)	102 (80.3%)
BMI (kg/m^2) on admission	22.9 (20.8-24.3)
TCHO on admission (mmol/L))	4.24 (3.70-4.94)
LDL on admission (mmol/L)	2.17 (1.82-2.59)
HDL on admission (mmol/L)	1.34 (1.14-1.65)
TG on admission (mmol/L)	1.31 (0.89-1.73)
Glucose on admission (mmol/L)	6.38 (5.66-8.10)
HbA1c on admission (%)	5.9 (5.7-6.5)

All values except for categorical data are represented as median (interquartile range).BMI, body mass index; HDL, high-density lipoprotein cholesterol; HbA1c, glycated hemoglobin; IQR, interquartile range; LDL, low-density lipoprotein cholesterol; n, number; TCHO, total cholesterol; TG, triglyceride

Table II. Characteristics of the overall patients (n=127)

Characteristic	Finding
Symptomatic, n (%)	59 (46.5%)
Statin user, n (%)	72 (56.7%)
History of hypertension, n (%)	111 (87.4)
History of diabetes mellitus, n (%)	44 (34.6)
MCA rSI	0.94 (0.84-1.03)
CASr (%)	75 (63.1-81.8)
MLD (mm)	1.1 (0.76-1.79)
wb-OEF	0.38 (0.33-0.42)
rCBF%	93.5 (86.9-100.8)
AI%	97.7 (94.3-100.6)

All values except for categorical data are represented as median (interquartile range). AI%, asymmetry index; CASr, carotid artery stenosis rate; IQR, interquartile range; MCA rSI, middle cerebral artery relative signal intensity; MLD, minimal luminal diameter; n, number; rCBF%, percentage of regional cerebral blood flow; wb-OEF, whole-brain oxygen extraction fraction

Table III. The relationship between the wb-OEF and the delay of the image count until the maximal filling of the contrast medium in the MCA distal to the carotid stenosis (n=127)

Count until maximal filling [image count (no. of patients)]	wb-OEF [median (IQR)]
0 (86)	0.36 (0.31-0.40)
1 (19)	0.41 (0.37-0.46)
2 (9)	0.42 (0.34-0.46)
3 (6)	0.41 (0.37-0.52)
4 (5)	0.47 (0.39-0.55)
5 (2)	0.45 (0.42-0.47)

All values are represented as median (interquartile range). MCA, middle cerebral artery; wb-OEF, whole-brain oxygen extraction fraction

Table IV. Differences in the wb-OEF between patients with count 0 and patients with count ≥1, based on the delay in the maximal filling in the MCA distal to the carotid stenosis

Count number (no. of patients)	Count number (no. of patients)	*P*-value
0 (86)	1 (19)	0.0010
0 (86)	2 (9)	0.0563
0 (86)	3 (6)	0.0995
0 (86)	4 (5)	0.0082
0 (86)	5 (2)	0.0513

MCA, middle cerebral artery; *P*, probability; wb-OEF, whole-brain oxygen extraction fraction

Table V. Differences in variables between patients in Group I and patients in Group 0

	Group I (N = 41)	Group 0 (N = 86)	*P*-value	*z* value
MCA rSI	0.83 (0.68-0.94)	1.0 (0.92-1.08)	<0.0001	-5.9
MLD (mm)	0.80 (0.57-1.05)	1.42 (1.00-2.08)	<0.0001	-5.1
CASr (%)	80.1 (76.5-88.0)	68.5 (60.3-80.2)	<0.0001	4.6
PSV (cm/s)	304 (169.5-382.5)	206.5 (106.6-268)	0.0035	2.9
rCBF%	90.7 (83.8-100.4)	95.4 (87.3-102.0)	0.0581	-1.9
AI%	97.1 (94.0-99.9)	97.8 (94.3-100.8)	0.2111	-1.2

All values are represented as median (interquartile range). Group 0 consists of patients with count 0; Group I consists of patients with count 1, 2, 3, 4, or 5. AI%, asymmetric index; CASr, carotid artery stenosis rate; IQR, interquartile range; MCA rSI, middle cerebral artery relative signal intensity; MLD, minimal luminal diameter; *P*, probability; PSV, peak systolic velocity; rCBF%, percentage of regional cerebral blood flow

Table VI. Spearman's rank correlation coefficients between MCA rSI, MLD, CASr, and PSV (n=127)

r_s	MCA rSI	MLD	CASr	PSV
MCA rSI		0.55	-0.49	-0.38
MLD	0.55		-0.94	-0.52
CASr	-0.49	-0.94		0.50
PSV	-0.38	-0.52	0.50	

CASr, carotid artery stenosis rate; MCA rSI, middle cerebral artery relative signal intensity; MLD, minimum luminal diameter; PSV, peak systolic velocity; r_s, Spearman's rank correlation coefficient

Table VII. The upper limits of the significant factors of Group I, using receiver operating characteristic curves of logistic regression analysis

	No. of patients	Sens (%)	Spec (%)	OR	*P*-value	AUC	BIC
MCA rSI	127						
(≤0.89 vs. >0.89)	45 vs. 82	70.7	81.4	0.139E-3 (0.285E-5 to 0.352E-2)	<0.0001	0.823	129
MLD	127						
(≤1.06 vs. >1.06) mm	59 vs. 68	80.5	69.8	0.127 (0.046-0.295)	<0.0001	0.782	140

BIC, Bayesian information criterion; MCA rSI, middle cerebral artery relative; signal intensity; MLD, minimal luminal diameter; OR, odds ratio; *P*, probability; Sens, sensitivity; Spec, specificity; vs., versus

Table VIII. Comparison between the combination of significant factors and Group I and Group 0

	Group I (N=41)	Group 0 (N=86)	OR	*P*-value	Chi-square value
				<0.0001	42.8
MCA rSI ≤0.89 and MLD ≤1.06 mm	27	10	24.3		
(MCA rSI ≤0.89 and MLD >1.06 mm) or (MCA rSI <0.89 and MLD ≤1.06 mm)	8	22	3.3		
MCA rSI >0.89 and MLD >1.06 mm	6	54			

Group 0 consists of patients with count 0; Group I consists of patients with count 1, 2, 3, 4, or 5.
MCA rSI, middle cerebral artery relative signal intensity; MLD, minimum luminal diameter; OR, odds ratio; *P*, probability

Table IX. Comparison between the significant predictor combinations and the whole-brain oxygen extraction fraction (n=127)

Combination of factors	No. of patients	wb-OEF	*P*-value	Chi-square value
			0.0032	11.5
MCA rSI ≤0.89 and MLD ≤1.06	37	0.41 (0.37-0.45)		
(MCA rSI ≤0.89 and MLD >1.06) or (MCA rSI >0.89 and MLD ≤1.06)	30	0.39 (0.32-0.42)		
MCA rSI >0.89 and MLD >1.06	60	0.36 (0.32-0.40)		

All values are represented as median (interquartile range). MCA rSI, middle cerebral artery relative signal intensity; MD, median; MLD, minimum luminal diameter; *P*, probability; wb-OEF, whole-brain oxygen extraction fraction

Table X. Comparison of the whole-brain oxygen extraction fraction between various combinations of significant factors (n=127)

Combination of factors	Combination of factors	*P*-value
MCA rSI ≤0.89 and MLD ≤1.06	MCA rSI >0.89 and MLD >1.06	0.0008
MCA rSI ≤0.89 and MLD ≤1.06	MCA rSI ≤0.89 and MLD >1.06) or (MCA rSI >0.89 and MLD ≤1.06)	0.0510
MCA rSI ≤0.89 and MLD >1.06) or (MCA rSI ≤0.89 and MLD >1.06)	MCA rSI >0.89 and MLD >1.06	0.3118

MCA rSI, middle cerebral artery relative signal intensity; MD, median; MLD, minimum luminal diameter; *P*, probability; wb-OEF, whole-brain oxygen extraction fraction

Table XI. Spearman's rank correlation coefficients between MCA rSI and rCBF% or between MCA rSI and AI% in patients with MCA rSI ≤0.89 and MLD ≤1.06 (n=37)

r_s	rCBF%	*P*-value	AI%	*P*-value
MCA rSI	0.4150	0.0107	0.5410	0.0005

AI%, asymmetric index; MCA rSI, middle cerebral artery relative signal intensity; MLD, minimal luminal diameter; *P*, probability; rCBF%, percent of regional cerebral blood flow; r_s, Spearman's rank correlation coefficient

Table XII. Upper limits of the MCA rSI for rCBF% <90% using ROC curves, based on logistic regression analysis in patients with MCA rSI ≤0.89 and MLD ≤1.06 (n=37)

	No. of patients	Sens (%)	Spec (%)	OR	*P*-value	AUC	BIC
MCA rSI (≤0.69 vs. >0.69)	11 vs. 26	47.4	88.9	1.87E-03 (1.79E-6 to 0.550)	0.0292	0.671	53.7

BIC, Bayesian information criterion; MCA rSI, middle cerebral artery relative signal intensity; MLD, minimum luminal diameter; OR, odds ratio; *P*, probability; rCBF%, percent of regional cerebral blood flow; Sens, sensitivity; Spec, specificity; vs., versus

Table XIII. Upper limits of the MCA rSI for AI% <90% using receiver operating characteristic curves, based on logistic regression analysis in patients with MCA rSI ≤0.89 and MLD ≤1.06 (n=37)

	No. patients	Sens (%)	Spec (%)	OR	*P*-value	AUC	BIC
MCA rSI (≤ 0.71 vs. > 0.71)	13 vs. 24	100	77.4	1.40E-07 (3.97E-14 to 1.65E-3)	0.0002	0.922	26.3

AI%, asymmetric index; BIC, Bayesian information criterion; MCA, middle cerebral artery; OR, odds ratio; *P*, probability; rSI, relative signal intensity; Sens, sensitivity; Spec, specificity; vs., versus
